# Assessing the potential for intraguild predation among taxonomically disparate micro-carnivores: marsupials and arthropods

**DOI:** 10.1098/rsos.171872

**Published:** 2018-05-02

**Authors:** Tamara I. Potter, Aaron C. Greenville, Christopher R. Dickman

**Affiliations:** 1 Desert Ecology Research Group, School of Life and Environmental Sciences, University of Sydney, Sydney, New South Wales, Australia; 2 National Environmental Science Programme Threatened Species Recovery Hub, University of Sydney, Sydney, New South Wales, Australia; 3 Long Term Ecological Research Network, Terrestrial Ecosystem Research Network, University of Sydney, Sydney, New South Wales, Australia

**Keywords:** intraguild predation, marsupials, micro-carnivores, Simpson Desert, spiders

## Abstract

Interspecific competition may occur when resources are limited, and is often most intense between animals in the same ecological guild. Intraguild predation (IGP) is a distinctive form of interference competition, where a dominant predator selectively kills subordinate rivals to gain increased access to resources. However, before IGP can be identified, organisms must be confirmed as members of the same guild and occur together in space *and* time. The lesser hairy-footed dunnart (*Sminthopsis youngsoni*, Dasyuridae) is a generalist marsupial insectivore in arid Australia, but consumes wolf spiders (*Lycosa* spp., Lycosidae) disproportionately often relative to their availability. Here, we test the hypothesis that this disproportionate predation is a product of frequent encounter rates between the interactants due to high overlap in their diets and use of space and time. Diet and prey availability were determined using direct observations and invertebrate pitfall trapping, microhabitat use by tracking individuals of both species-groups, and temporal activity using spotlighting and camera traps. Major overlap (greater than 75% similarity) was found in diet and temporal activity, and weaker overlap in microhabitat use. Taken together, these findings suggest reasonable potential, for the first time, for competition and intraguild predation to occur between taxa as disparate as marsupials and spiders.

## 1. Introduction

As resources are limited across space and time, species often exploit different niches or partition shared resources [1–3]. However, in variable environments where resource availability can fluctuate spatially and temporally, or for members of an ecological guild, species frequently compete for access to the same resources [4–6]. An ecological guild groups species if they share resources or exploit them in a similar manner, irrespective of their phylogenetic relationships [4,6–9]. For instance, insectivores and frugivores exemplify foraging guilds, while arboreal or fossorial animals represent habitat or microhabitat guilds [5,10]. The guild concept was developed in relation to competition theory, with interspecific competition presumed to be more intense between members of the same guild and weaker or non-existent between members of different guilds [7,11–13].

Competition manifests in a number of forms. A particularly distinctive type—interference competition—occurs frequently among species in the carnivore guild and is characterized by aggressive interactions [14,15]. Intraguild predation (IGP) can be seen, in turn, as a unique and extreme form of interference competition, where a dominant predator selectively kills and eats subordinate rivals to gain increased access to resources [14,16,17]. For example, in Australian systems the larger and dominant dingo (*Canis dingo*) will kill and sometimes consume the invasive red fox (*Vulpes vulpes*), thereby reducing competition for shared prey [18,19]. These interactions are usually asymmetric and facilitated by differences in body size, with killing often arising when the larger carnivore is 2–4 times the size of the subordinate [14,17]. Additionally, at times or in environments where prey is scarce, antagonistic encounters are likely to escalate [14].

Before IGP can occur, several preconditions should be satisfied. Firstly, and most importantly, any two species involved in the interaction must belong to the same resource guild, specifically a carnivorous foraging guild (e.g. they should be insectivores or apex predators). Another prerequisite is syntopy; that is, the species in question must occur together in both space *and* time [4,7,9,12]. As different factors govern the distribution and abundance of species at different spatial resolutions, it is important also to consider the scale most appropriate to investigate these interactions [20,21]. For example, at a broad scale, climate and topography are likely to be important determinants of distribution, while at a finer scale, food availability and inter- and intraspecific interactions may be more crucial [20,22]. Quantifying habitat at a fine scale, such as the shared use of certain microhabitats, is required to assess IGP. In comparison to macrohabitat, which is often defined as the dominant vegetation formation (e.g. forest, grassland), microhabitat refers to the structural components within a given habitat, such as open patches in a forest [21,23]. Temporal overlap in activities is also critical for IGP; predation can occur only if interactants are active at similar times [1,3]. Akin to documenting habitat selection at a fine spatial scale, temporal activity therefore should also be examined if the potential for IGP is to be appraised. IGP may develop simply as a consequence of encounter rates escalating with increased overlap in niche use [24,25].

Despite its prevalence within guilds of carnivores, most examples of IGP have been reported between taxa that share phylogenetic similarity; few examples of IGP have been described between species from different phyla [6,26,27]. Recent studies investigating the diet of an insectivorous marsupial, the lesser hairy-footed dunnart, *Sminthopsis youngsoni* (Family Dasyuridae), have found that wolf spiders (Family Lycosidae) were consumed disproportionately often relative to their availability and to that of other spiders [28]. Reasons for this selective predation remain unclear, but investigation into the composition of various prey types in the diet of *S. youngsoni* has revealed that lycosids are unlikely to be targeted for nutritional reasons; levels of water, energy and nutritional composition vary little between lycosids and other invertebrates that are eaten by *S. youngsoni* [29]. Rather, alternative factors, such as high encounter rates between lycosids and *S. youngsoni*, may drive the observed selectivity. Both faunal groups are abundant and widespread in arid Australia, including in the Simpson Desert where the present study was carried out, and occupy a wide array of microhabitats [30–33]. Additionally, both *S. youngsoni* and lycosids are predominantly nocturnal and have been classified as generalist insectivores [34–36]. Given these similarities, the potential for fine-scale dietary, spatial and temporal overlap is relatively high.

This study asks whether the key requirements of competition and IGP prevail in the interaction between syntopic lycosids (*Lycosa* spp.) and *S. youngsoni*. We predicted initially that *S. youngsoni* would exhibit greater overlap in diet with lycosids than with other common invertebrate predators. From direct observations in the study region, results from invertebrate pitfall trapping (see below) and published work [37], the only invertebrate predators present in sufficient numbers to warrant comparisons were prowling spiders (Family Miturgidae). Based on results from these comparisons, we then investigated overlap in activity and microhabitat use between *S. youngsoni* and lycosids, and predicted that overlap will be high enough to assign these two taxa to the same foraging guild. We use the findings to assess the potential for IGP between the lycosid and marsupial predators.

## 2. Methods

### 2.1. Study site

**Figure 1.**
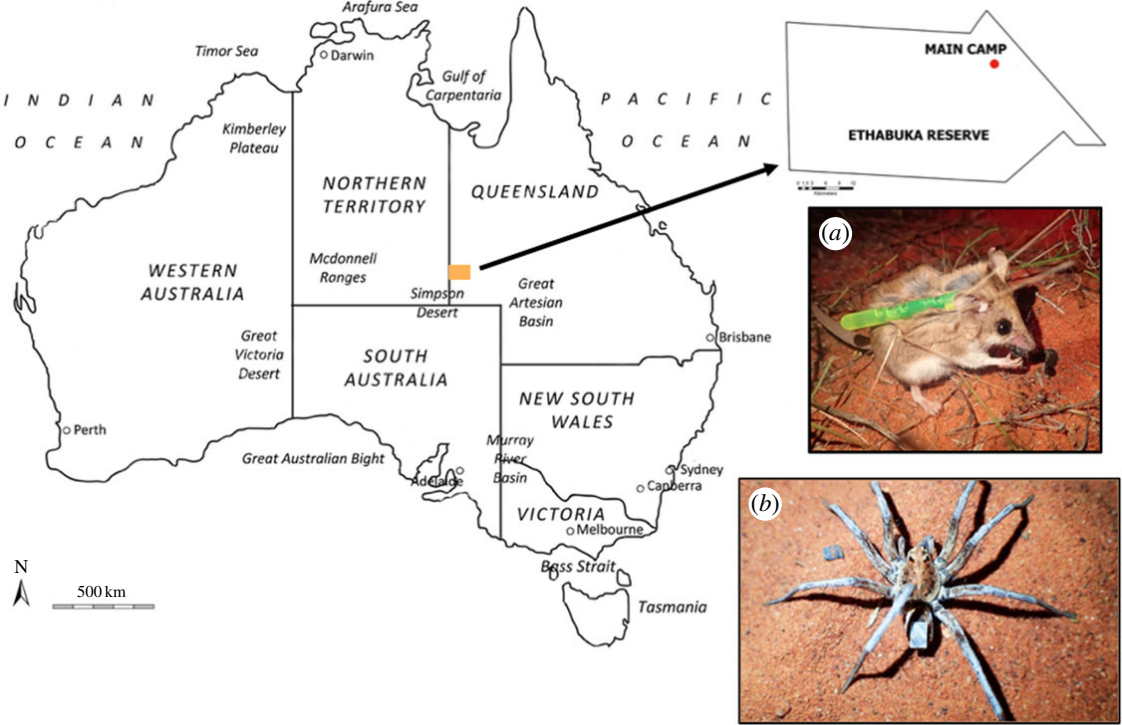
Location of Main Camp field site on Ethabuka Reserve (23°46′ S, 138°28′ E, inset) in the northeastern corner of the Simpson Desert, Queensland (orange rectangle), with images of both study species and methods of tracking also shown: (*a*) a small, non-toxic light stick (4.5 mm diameter × 39 mm long) was secured to the nape of individual *Sminthopsis youngsoni* (*n* = 10) to facilitate direct observations of prey selection, while (*b*) a square of reflective tape (3 × 3 mm) was secured to the opisthosoma of captured lycosids (*Lycosa* spp., *n* = 17) to facilitate observations and reveal patterns in microhabitat use and prey capture.

Observations were made at Main Camp, Ethabuka Reserve (23°46′ S, 138°28′ E), in the northeastern Simpson Desert, Queensland (figure 1), during three field trips in April, July and October 2016. The Simpson Desert covers 170 000 km^2^, with 73% of the region characterized by long, parallel sand dunes which run north-northwest to south-southeast [38–40]. The dunes are 0.6–1 km apart and reach elevations of 8–10 m [37,41,42]. Other landscape features include clay pans, rocky outcrops and gibber flats [38,43]. The dominant vegetation is spinifex (*Triodia basedowii*) grassland; however, the relatively open dune crests have a patchy cover of shrubs, perennial and ephemeral plants [37,44]. Small stands of gidgee trees (*Acacia georginae*), mallee eucalypts and other *Acacia* shrubs occur on the heavier clay soils of the interdune swales [37,39,45].

The daily maximum temperatures during summer in the Simpson Desert often exceed 40°C, while minima in winter drop below 5°C at night [41]. The study site lies within the 100 and 150 mm median annual rainfall isopleths, with most falls occurring during summer (December–March) [37,39,41]. However, heavy rains can descend at any time, locally or regionally [37,46,47]. During 2016, a negative Indian Ocean Dipole, a La Niña-like Pacific Ocean and very warm waters north of Australia combined to make the period between May and September one of the wettest on record for central Australia [48].

### 2.2. Capture of study species

In order to quantify resource use and activity patterns, dunnarts and individuals from both spider families (*Lycosa* spp.: Lycosidae, and Miturgidae) were live-captured, marked and then released at the point of capture to assess diet and microhabitat preferences.

Individual *S. youngsoni* were live-captured in pitfall traps on 16 trapping grids located 0.6–2 km apart at Main Camp during July and October 2016. Each grid comprised 36 pitfall traps in a 6 × 6 formation with traps set 20 m apart [49]. Grids encompassed all dune zones (crest, side and swale) and covered 1 ha [49,50]. A trap consisted of a PVC pipe 60 cm deep × 16 cm diameter, dug flush with the sand surface and overlain by a 5 m long, 300 mm high drift fence of aluminium flywire to increase trapping efficiency [51,52]. Traps had a wire mesh floor to prevent animals from digging out and also permitted water to drain freely in the event of rain [20]. Traps remained open for three consecutive nights and were checked once or twice a day to remove animals and reduce capture-stress [51]. Captured individuals were identified, weighed, sexed and reproductive status checked, and then given a unique ear clip [20,49,51]. Each individual was held by day in a plastic terrarium with shelter (calico bag) and food (mealworms) provided. Terrariums were placed in the shade to minimize heat exposure and encourage dunnarts to resume their natural diurnal resting pattern.

Lycosid and miturgid spiders were collected opportunistically from vertebrate pitfall traps (see above) or through active searches at night at Main Camp. Details regarding capture of invertebrates can be found in Potter [29]. Upon collection, spiders were weighed, identified to family, and sex determined from inspection of pedipalp size and morphology [34,36]. The taxonomy of spiders in both families is not well resolved, but our samples of lycosids appeared to comprise two similar morphs within *Lycosa*. Spiders were kept under shade in individual plastic vials until nightfall.

### 2.3. Diet and dietary overlap of predators

In order to confirm the assignation of the two species-groups (*S. youngsoni* and lycosids) to the same foraging guild and test the hypothesis of dietary similarity, several methods were employed to quantify diet composition and overlap. Direct observations were used to determine diet, and invertebrate pitfall traps to evaluate prey availability.

Captured *S. youngsoni* were released and followed to directly observe their prey selection. After dusk, a small non-toxic glow stick, 4.5 mm diameter × 39 mm long (Neptune Tackle, Adelaide, South Australia) was activated before being secured to the hair of a dunnart from the nape down the back using non-toxic cyanoacrylate glue (Selley's Quick Fix ‘superglue’) and positioned so as not to impede head or leg movement [53] (figure 1*a*). The dunnart was released approximately 10 m from the trap it was captured in, then observed until it was either lost from sight (e.g. in a burrow), became inactive, or the light emitted from the glow stick faded. Distance of the observer to the dunnart varied depending on the microhabitats encountered, the speed of the dunnart, and the activity the dunnart was engaged in. Red light was used to illuminate dunnarts when foraging to facilitate identification of selected prey, minimize disturbance to their natural foraging behaviour, and to preserve each observer's night vision [54]. Numbers, type and size (seven size classes were used, see below) of prey eaten were recorded, as well as time of night and method of prey capture. Dunnarts were not recaptured at the end of the observation period as glow sticks were expected to fall off with shedding fur within a few days [55,56], and chasing them may have caused unnecessary stress.

To quantify foraging and prey selection by lycosid and miturgid spiders, we scored the type and size of all prey items depredated during observations (see below). Prior to release, a 3 × 3 mm square of silver reflective tape (Class 1 reflectivity, Seton Australia) was attached to the opisthosoma (figure 1*b*) using non-toxic cyanoacrylate glue to increase the detectability of spiders when tracking them in low light conditions. Addition of reflective tape did not affect microhabitat use or activity [29]. Similar-sized individuals were selected for observations (mass ± s.e.; 0.49 ± 0.036 g and 0.51 ± 0.02 g for lycosids and miturgids, respectively), as a positive relationship exists between predator body size and the mean body size of invertebrate prey [35,57]. Spiders were released near their capture site between 20.00 h and 23.00 h around Main Camp, and followed and observed for approximately 1 h under red torchlight at a distance of 2–3 m to minimize disturbance. If we observed prey being captured, we would carefully approach to within approximately 1.5 m to confirm its size and identity.

Along with direct observations, invertebrate pitfall trapping was employed to quantify food availability and selectivity for both spiders and *S. youngsoni*. Pitfall traps were deployed every 5 m along dunnart movement trails and randomly oriented control paths, and at every third flag along actual and control paths of spiders (see §§2.5.1 and 2.5.2). A trap comprised a plastic vial (40 mm diameter × 100 mm deep) filled with approximately 80 ml of 3% formalin solution [20,32]. Pitfall traps were buried flush with the soil surface to maximize capture efficiency and left *in situ* for three nights [37]. A minimum of three vials was deployed along the trails taken by each dunnart and spider, as well as along their corresponding control trails. Lids were placed over open traps each morning and removed late each afternoon to ensure that only nocturnal invertebrates were collected. Thus, sampling was intended to reflect the actual prey types potentially available to dunnarts and spiders.

Pitfall trapping is limited with respect to the sampling of some invertebrates, such as large terrestrial and aerial species [20]. However, as aerial species are largely unattainable to ground-active spiders and *S. youngsoni*, and as these invertebrates form a negligible component of the diet of this dunnart [31,58,59], pitfall traps may still be suitable for providing an index of food availability [20,31]. Additionally, use of pitfall traps in conjunction with direct observations of foraging increased our ability to gain deep understanding of prey selection and diet of the study organisms.

After collection from the field, invertebrate pitfall vials were rinsed in water and 80% ethanol before inspection [37]. Invertebrates were identified to Order, except for spiders (Order Arachnida) and bees, wasps and ants (Order Hymenoptera) which were identified to Family using identification keys [60,61]. Invertebrates were identified to these levels as knowledge of finer-scale taxonomy for many arthropods in the study region is lacking [62,63]; therefore, confident species- and genus-level identification for many taxa was not feasible [37].

Invertebrates were counted and grouped into seven size classes (Class I = <2 mm, Class II = 2–4.9 mm, Class III = 5–6.9 mm, Class IV = 7–9.9 mm, Class V = 10–14.9 mm, Class VI = 15–19.9 mm, Class VII = >20 mm) based on total body length from head to abdomen, with the exclusion of appendages (antennae, legs) to determine if the predators partition prey based on size [20,37].

#### 2.3.1. Statistical analyses: diet and dietary overlap

The numbers of captures of each prey taxon were recorded for *S. youngsoni*, lycosids and miturgids during observations, and these values were used to calculate trophic diversity using Simpson's [64] diversity index, *D* (equation (2.1)) [65]:

2.1 $$D=\frac{\left({\left[\Sigma {P_{i}}^{2}\right]}^{-1}-1\right)}{\left(n-1\right)},$$

where *P_i_* is the proportion of the *i*th prey taxon captured by a predator. The inverse of this index was employed so that *D* increases from 0 (low diversity) to 1 (high diversity), thus providing a measure of dietary specialization [10,66,67]. Raw capture values were converted into proportions relative to total numbers of prey for each prey category, before quantifying overlap between *S. youngsoni* and the two spider families using the symmetrical version of MacArthur & Levin's [68] and Pianka's [69] overlap equation (2.2):

2.2 $$O_{jk}=\frac{\Sigma P_{ij}P_{ik}}{\sqrt{(\Sigma \;{P_{ij}}^{2}\Sigma \;{P_{ik}}^{2})}},$$

where *O_jk_* is the measure of overlap between species *j* and species *k*, and *P_ij_* and *P_ik_* refer to the proportions of resource *i* used of the total resources used by species *j* and *k*, respectively. This measure of overlap can vary between 0, i.e. no resources are used in common, to 1, complete overlap [70,71], with values greater than 0.75 generally representing strong overlap [26,72,73]. This index is sensitive to the number of prey categories and sample sizes used [70,74] but, due to its simplicity, it has been widely used in the literature [71,75], thus enabling broad ecological comparisons to be made.

To test whether overlap values differed from chance, observed values were compared against a simulated null distribution using ‘EcoSimR’ [76] in R v. 3.2.2 [77]. One thousand model runs were used to generate a simulated mean, with 95% confidence intervals (CI) and associated *p* values. Overlap in prey sizes eaten by lycosids, miturgids and *S. youngsoni* was calculated as for overlap in prey taxa, and using the same seven categories of prey length employed for pitfall trap-sampled invertebrates (see above) as a measure of prey size. As before, these values were also compared against simulated distributions.

Counts for each arthropod prey taxon sampled along the movement and control paths of individual dunnarts and spiders were pooled, as the invertebrate pitfall traps along these paths were not statistically independent. Additionally, due to the short distances (less than 5 m) between these traps, captures in one trap may preclude captures in an adjacent trap [37,78]. Pooling also minimized fine-scale disparities in catch size between adjacent traps and allowed an index of mean prey availability to be calculated for each predator [37,79]. Counts of prey within each size category were tallied per trail. Total numbers of arthropods sampled (all Orders combined) were pooled for each trail and then averaged across individuals for each species and trail type. A two-way analysis of variance was employed to test whether total numbers of arthropods differed between trail type and species, using SPSS v. 24 [80]. Assumptions of normality and homogeneity of variance were checked; as normality was not met, data were log(*x* + 1) transformed prior to analyses [81].

To test whether assemblages of arthropod prey differed between the two trail types (control and actual animal movement paths) for both lycosids and *S. youngsoni*, non-metric multidimensional scaling (nMDS) ordinations were run using e-PRIMER v. 6.1 [82,83]. Data were fourth-root transformed to reduce the influence of common prey taxa (e.g. ants) [84,85]. Additionally, due to small numbers of captures, Lepidoptera (moths) and Orthoptera (grasshoppers) were combined into the category ‘Other’ for each trail, and one *S. youngsoni* control trail was removed prior to analyses as it had no prey captures. A Bray–Curtis similarity matrix was calculated and vectors for each arthropod taxon were fitted to the ordination, with vector length proportional to Spearman's correlation between the variable and the ordination [83,86].

Following this, non-parametric permutational analysis of variance (PERMANOVA) was used to test for differences in prey taxa and prey sizes between trail type (fixed factor, 2 levels: control and actual animal movement path) for *S. youngsoni* and lycosids independently [82,87]. Similarly, to determine if foraging paths of the two predator species-groups had dissimilar prey availability, prey abundance and size categories were compared along actual trails taken by *S. youngsoni* and lycosids. PERMANOVAs were run using Bray–Curtis similarity matrices and 9999 permutations. PERMANOVA is sensitive to heterogeneity of multivariate dispersion among groups [88], so this assumption was tested using PERMDISP, a multivariate extension of Levene's test [82,89]. See electronic supplementary material, table A1, for results. These multivariate analyses were executed using e-PRIMER v 6.1 with the PERMANOVA+ v. 1.0.6 add-on package [82,83].

### 2.4. Temporal activity and temporal overlap of predators

As dietary analyses revealed high overlap between *S. youngsoni* and lycosids, but weak overlap between *S. youngsoni* and miturgids, combined with the fact that *S. youngsoni* selectively depredates lycosids, temporal and spatial overlaps were quantified only between lycosids and *S. youngsoni*.

To quantify the temporal activity of lycosids, spotlight surveys were conducted in October 2016 every hour between dusk (19.30 h) and dawn (05.30 h) over three nights. This yielded 33 transect surveys. Each hour, a 100 m transect was walked for 10 min using a spotlight (Fenix TK35, 960 lumens) to detect lycosid eye shine [90]. For consistency, each survey was conducted along the same 100 m transect, which was marked by a row of six remote cameras (see below). A transect was used rather than a random walk to ensure varied microhabitats were surveyed (including spinifex hummocks and open sand) and to reduce bias towards open areas where walking was easier and spiders more easily detected. Numbers of spiders observed in each 10 min survey were tallied.

Remote camera traps were deployed to further quantify patterns in the activity of both spiders and dunnarts. Twenty-four Reconyx PC800 Hyperfire™ cameras (Reconyx, Inc., Holmen, WI, USA) were deployed on 7 July 2016 at Main Camp and left until 12 October 2016 (98 days, or 2352 h of deployment). Cameras were placed on dune crests and in swales, as well as in burnt and unburnt areas, to get a complete representation of activity across the entire dune system. Based on pilot trials, half the cameras were positioned vertically and half angled at approximately 45° to the ground. Cameras angled at 45° had a greater field of view and were more likely to detect *S. youngsoni*, while those facing down had more chance of detecting lycosids. Cameras were placed along four north–south facing 100 m transects with six cameras per transect each spaced 20 m apart. Cameras were attached to metal posts approximately 50 cm above the ground surface. To increase lycosid capture success, cameras were set to take both time-lapse and motion-triggered images. Settings were as follows: time-lapse—single image every 5 min between 19.00 h and 07.00 h, and motion-trigger—single image with no delay between triggers (i.e. rapid-fire) and sensitivity set as high to maximize detection rate.

Each image was tagged with location (burnt or unburnt), position (crest or swale), camera angle (angled or vertical), camera ID number, species and confidence level (‘possible’, ‘probable’ and ‘definite’), and the tags written to the EXIF data of each file using the multi-format graphics program XnView MP v. 0.83 [91]. ‘EXIF data’ refers to the information stored within an image and can include GPS location, camera make and model, time and date, exposure, shutter speed and resolution. All fauna groups were tagged and a reference library was built containing each camera-detected species to assist identification.

#### 2.4.1. Statistical analyses: temporal activity and overlap

EXIF data, including date, time and tagged keywords, were extracted from each image and written to an Excel file using the command line package ‘exiftool’. To ensure independence, multiple photographs likely to be of the same individual (photographs in sequence less than 2 min apart) were removed prior to analysis [43,92]. To determine activity patterns of lycosids and *S. youngsoni*, photographs were pooled across all cameras, habitat types, locations and positions [43]. Images with ID confidence tags of ‘definite’ and ‘probable’ were used for analyses (51 images of *S. youngsoni* and 304 images of lycosids). As activity times follow a circular distribution over 24 h, mean activity times and confidence intervals were calculated for both species using the circular statistics program Oriana v 4.02 [93]. Lycosid mean activity times and confidence intervals were also calculated from spotlighting data using this program.

To assess overlap in nocturnal activity patterns between lycosids and *S. youngsoni*, and thus the potential for competition and predation, the ‘Overlap’ v. 0.2.7 package [94] in R v. 3.2.2 [77] was used. This software fits kernel density curves to observation times for a particular species and estimates the degree of overlap between species [94]. This ‘coefficient of overlapping’ is a quantitative measure that ranges from 0 (no overlap) to 1 (complete overlap) [95–97]. Bootstrapping was also applied to calculate 95% confidence intervals and to test whether estimated coefficients of overlapping were drawn from a random sampling distribution [95]. Prior to analysis, times were converted to radians as density curves are fitted using trigonometric functions [96]. Kernel density fitting was also applied to spotlighting data.

### 2.5. Microhabitat use and microhabitat overlap of predators

#### 2.5.1. Dunnarts

To quantify the degree of microhabitat selectivity displayed by *S. youngsoni*, movement patterns of captured individuals were quantified using spools and lines (*n* = 26, 15 in July and 11 in October 2016). Pregnant females or those with joeys were excluded. Prior to release, a 2-ply cotton bobbin spool (Coats Australia Pty, Sydney, Australia) was secured using the same method for attaching glow sticks (§2.3 and figure 1*a*). Spools were adjusted to weigh approximately 6% of individual body mass (mean ± s.e.; 0.65 ± 0.18 g) and secured with tape [55]. This spool mass was used as it does not impede maximum running speed [98,99], especially in females, which are accustomed to carrying young [100]. Moreover, animals emerge and recommence normal activity after an initial flight response [98] while carrying spools of this size. Individuals were released with spools within 1–3 h of dusk as this is when they are most active [53]. The end of the spool was tied to a sturdy plant stem, approximately 10 m from where each animal was captured, and dunnarts were observed for a few minutes to ensure that release was successful. Each individual was tracked once only.

The following day, spool lines were followed and the cumulative distance travelled by each released animal measured to the nearest 0.1 m using a tape measure [32]. Additionally, percentage cover of seven different microhabitat types was estimated visually using a 0.5 × 0.5 m quadrat at fixed 2.5 m intervals along the spool trails [23,32,101]. These microhabitats were: live spinifex, dead spinifex, ground cover, shrub cover, dead wood, all other vegetation, and bare ground (table 1). These variables were selected as prior research has found them to influence the distribution and abundance of dasyurids [32,102]. Distance to nearest cover was recorded, and the first 5 m of each trail excluded as an initial flight response [50,103]. Spools were removed from any dunnarts that were recaptured, otherwise empty spool cases were expected to fall off within a few days [55].

**Table 1.** Habitat characteristics scored along the actual movement trails, and control (random) trails, of captured *Sminthopsis youngsoni* and *Lycosa* spp., Simpson Desert, southwestern Queensland.

habitat variable	unit	description
spinifex (alive) cover	%	total cover of alive (green) spinifex hummocks (including tips)
spinifex (dead) cover	%	total cover of horizontal brown, burnt or dry spinifex attached to live spinifex or rooted in the ground
all other vegetation cover	%	total cover of all vegetation, excluding spinifex (e.g. *Aristida* spp., *Yakirra* spp.) that can provide cover for fauna
ground cover	%	total cover of vegetation that does not provide cover, including leaf litter, senesced leaf foliage, small detached woody debris less than 1 cm diameter (e.g. twigs), sparse seedlings
shrub cover	%	total cover of live woody plants with foliage
dead wood cover	%	total cover of logs (diameter >1 cm) and attached or detached dead woody plants without foliage
bare ground cover	%	total cover of bare or cryptogamic soil (cryptogam = biological soil crust, e.g. composed of cyanobacteria, fungi or lichens)
distance to cover	cm	distance from spool or control line to the nearest cover (for *S. youngsoni*—diameter >20 cm, height >10 cm; for lycosids—height >5 cm, diameter >5 cm). Cover was deemed to be any vegetation or dead wood where a dunnart or lycosid could completely conceal its body from a predator

In order to assess the degree of microhabitat selectivity exhibited by *S. youngsoni*, a single control trail for each actual spool trail left by a dunnart was also scored as a measure of the availability of each microhabitat within the local environment [32]. A control trail began at the starting point of an associated spool trail and followed a straight line along a randomly selected compass bearing [32]. Control trails were set to be the same length as actual spool trails and microhabitat scoring was consistent with that employed for spool trails, thus presenting a standardized method for comparison between the two trail types.

#### 2.5.2. Spiders

We assessed microhabitat use and selectivity of lycosids during observations of their foraging behaviour (§2.3). A flag was deployed at the start of each spider's trail and then at approximately 2.5 m intervals to record the path taken, with a total of 12 flags deployed per trail. Spiders were observed for approximately 1 h or until all 12 flags were deployed. The next day, trail length was measured to the nearest 0.1 m and percentage cover of microhabitat variables estimated using a 0.5 × 0.5 m quadrat at each flag. The same variables recorded along *S. youngsoni* trails were scored so that comparisons could be made between the two taxa (table 1) [23]. Quadrats were centred on the flags and distance to nearest cover was measured from this centre point. As with *S. youngsoni*, a control trail was also scored to measure availability of these microhabitats within each individual's local environment [32].

#### 2.5.3. Statistical analyses: microhabitat use and overlap

Measurements of each microhabitat variable were averaged along each spool and control line so that replication was the number of individual animals tracked. To visualize patterns of resemblance between the two trail types (control and spool) and season (winter and spring) for both lycosids and *S. youngsoni*, nMDS ordinations were constructed using e-PRIMER v. 6.1 [82,83]. The same procedures as used for diet analyses were used here, with minor deviations. As well as being fourth-root transformed to reduce the impact of common microhabitat structures (e.g. spinifex), data for each variable were normalized independently as microhabitat variables were measured on different scales (percentages and centimetres). Normalization involved subtracting the mean for each variable and dividing by one standard deviation [83]. As the resulting matrix contained negative values, nMDS ordinations were based on Euclidean distances. As with ordination for arthropod pitfall data, vectors for each microhabitat variable were fitted to the ordination [86].

Non-parametric PERMANOVAs were run to test for differences between trail type (control or actual) and season (winter and spring) for both lycosids and *S. youngsoni* and to compare habitat along trails travelled by the two species-groups [82,87]. For each species-group, trail type was nested within season (data were collected only once per season). PERMANOVAs were run using 9999 permutations, following testing for heterogeneity of multivariate dispersion using PERMDISP [82,89]. For results see electronic supplementary material, table A2. Similarity percentages (SIMPER) analysis was also used to determine which habitat variables contributed most to the observed pattern of separation between lycosids and *S. youngsoni* [81,83].

## 3. Results

### 3.1. Prey selection, diet and dietary overlap

A total of 10 *S. youngsoni* was followed during October 2016, May and October 2017, with prey capture witnessed on 13 occasions. Total time spent observing this species was 4.2 h. In comparison, the time spent observing spiders (lycosids and miturgids) was 27 h (approx. 6.5 h in April, 1 h in July and 19.5 h in October 2016), with 17 individual lycosids and 10 miturgids observed, and totals of 30 and 13 direct prey captures recorded, respectively. Prey taxa consumed most often by lycosids were ants (23% of observations), spiders (20%, both inter- and intraspecific) and moths (17%), while prey consumed most often by *S. youngsoni* were ants and spiders (74% combined). Lycosids constituted 13% of all captures by *S. youngsoni,* while no miturgids were observed being consumed by this species*.* Moths were selected most often by miturgids (38%), while the only spiders consumed by miturgids were jumping spiders (7% of captures).

In general, diversity of prey types consumed was low for all species. Miturgids exhibited the most diverse diet with a Simpson's diversity index of 0.42. Simpson's diversity was 0.28 and 0.18 for lycosids and *S. youngsoni*, respectively. A value of 0.83 was obtained using Pianka's overlap index for prey species captured by *S. youngsoni* and lycosids, with this observed value falling within the top 2.5% of the simulated distribution (mean = 0.43, 95% CI = 0.15–0.76, *p* = 0.002). By contrast, the observed overlap value of 0.39 between *S. youngsoni* and miturgids was not distinguishable from random (mean = 0.35, 95% CI = 0.05–0.89, *p* = 0.35), with a similar trend for the observed overlap index of 0.68 between lycosids and miturgids (mean = 0.52, 95% CI = 0.26–0.84, *p* = 0.24).

Observed overlap in prey sizes was 0.68 between *S. youngsoni* and lycosids, 0.57 between *S. youngsoni* and miturgids, and 0.91 between lycosids and miturgids. These values did not differ from the simulated mean for *S. youngsoni* and lycosids (mean = 0.46, 95% CI = 0.14–0.93, *p* = 0.20) or for *S. youngsoni* and miturgids (mean = 0.58, CI = 0.04–0.95, *p* = 0.12), but overlap between lycosids and miturgids was statistically higher than by chance (mean = 0.38, CI = 0.08–0.89, *p* = 0.02).

**Figure 2.**
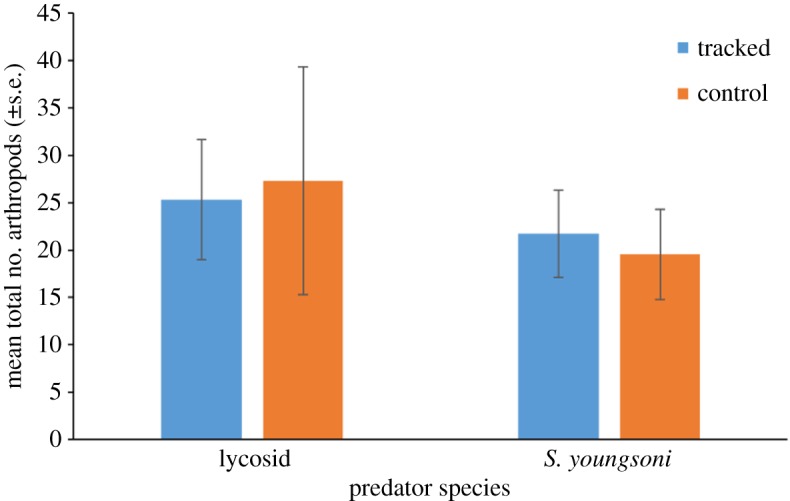
Total number of arthropods sampled in pitfall traps deployed along control (random) trails and paths travelled by spooled dunnarts and tracked lycosids at Main Camp, Simpson Desert, southwestern Queensland in October 2016. Numbers of arthropods were summed for each individual trail and then averaged (s.e.) for each trail type and species. Analysis of variance revealed no significant difference between trail type (*F*_1,41_ = 0.791, *p* = 0.379), species (*F*_1,41_ = 0.008, *p* = 0.931) or in the interaction between these two factors (*F*_2,8_ = 0.005, *p* = 0.946).

In total, 12 113 arthropods were sampled from pitfall traps, with ants accounting for 93% of this total. A total of 31 spiders were captured across all pitfall traps, 14 of these were miturgids. Mean overall abundance of arthropods did not differ significantly between trail type (*F*_1,41_ = 0.791, *p* = 0.379), species (*F*_1,41_ = 0.008, *p* = 0.931) or in the interaction between these two factors (*F*_2,8_ = 0.005, *p* = 0.946; figure 2).

**Figure 3.**
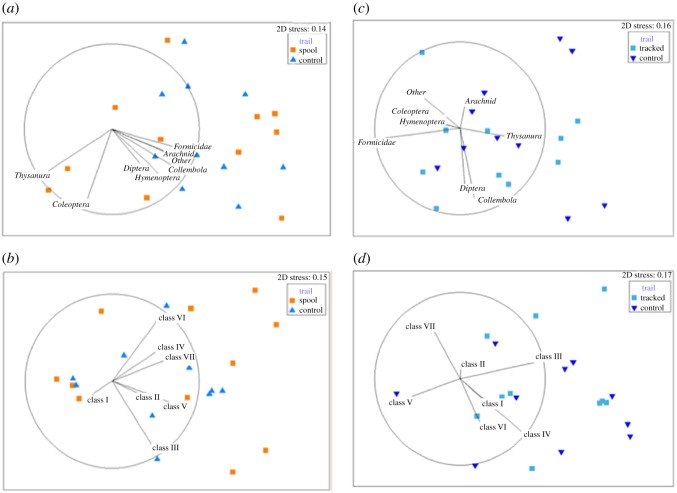
Non-metric multidimensional scaling ordination plots for arthropod (*a*) taxa and (*b*) size classes sampled in pitfall traps deployed along actual spool and control lines for *Sminthopsis youngsoni* and (*c*) taxa and (*d*) size classes sampled along actual lycosid tracks and control lines at Main Camp, Simpson Desert, southwestern Queensland, in October 2016. Data were fourth-root transformed before ordination was performed on Bray–Curtis similarities. Stress values are less than 0.2, indicating that similarity between data points in 2D is reliable. Vectors for each variable (arthropod taxon or size class) were fitted to the ordination, with vector length proportional to the Spearman's correlation between the variable and the ordination, and direction indicating the gradient of each variable. Taxa were as follows: Arachnids (spiders, mites, pseudoscorpions), Coleoptera (beetles), Collembola (springtails), Diptera (flies), Hymenoptera (bees, wasps) and ‘other’ (grasshoppers and moths combined). See main text for distinctions between size classes.

**Figure 4.**
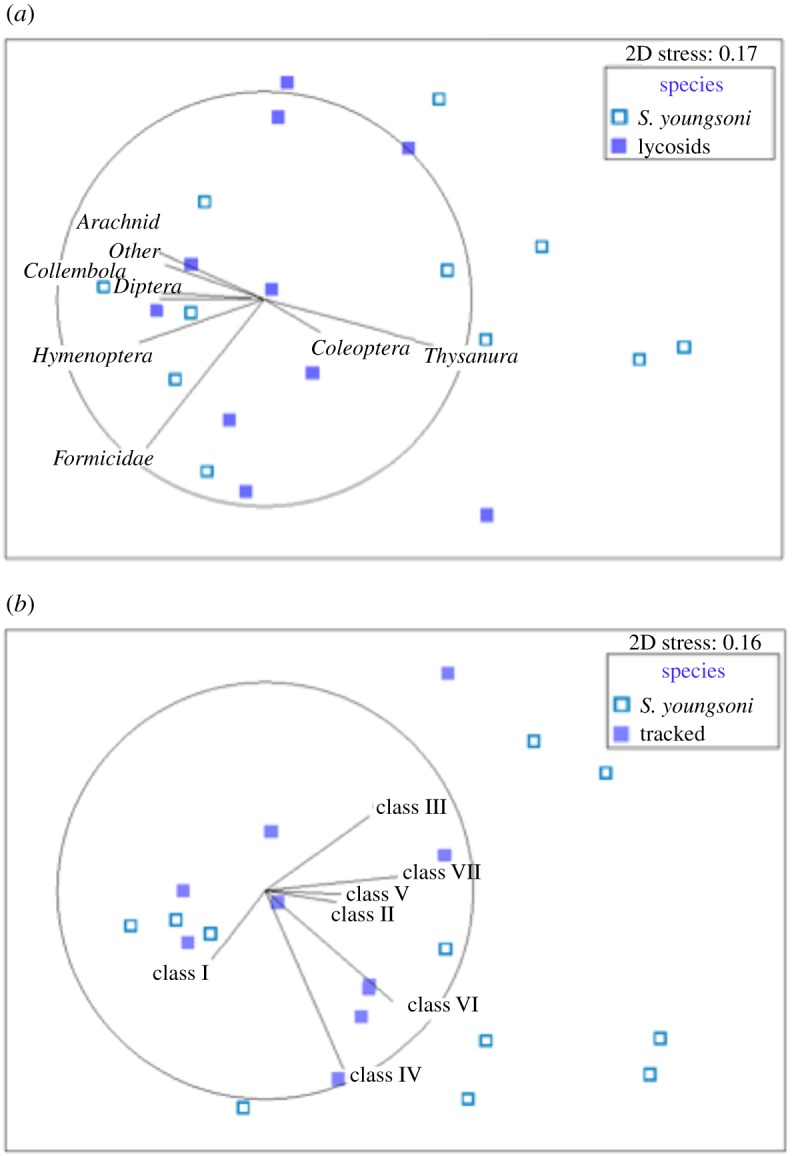
Non-metric multidimensional scaling ordination plots for arthropod (*a*) taxa and (*b*) size classes sampled in pitfall traps deployed along paths travelled by tracked lycosids and *Sminthopsis youngsoni* at Main Camp, Simpson Desert, southwestern Queensland, in October 2016. Data were fourth-root transformed before ordination was performed on Bray–Curtis similarities. Stress values are less than 0.2, indicating that the similarity between datapoints represented by this ordination is reliable. Vectors for each variable (arthropod taxon or size class) were fitted to the ordination, with vector length proportional to the Spearman's correlation between the variable and the ordination, and direction indicating the gradient of each variable. See main text for distinctions between size classes.

Multidimensional scaling produced no clear separation between control and actual spool trails in numbers of arthropod taxa or prey size classes sampled for *S. youngsoni* (figure 3). Two spool trails, correlated with Thysanura (silverfish) and Coleoptera (beetles), differed from a cluster containing all other trails (figure 3*a*). With respect to prey size, the trajectory of size Class I differed from all other size classes (figure 3*b*). Results for lycosid trails were similar, with no clear distinction between prey taxa or size classes sampled along either control or actual lycosid trails. For prey taxa, most datapoints aligned with Thysanura, Collembola and Diptera (figure 3*c*), whereas for size classes a contrast is apparent between an outlier from a control trail associated strongly with Class V and all other datapoints (figure 3*d*). When comparing the paths travelled by each species-group (*S. youngsoni* and lycosids), no distinct separation is evident for either prey taxa or size class (figure 4). Stress values for all plots were less than 0.2, indicating that these two-dimensional (2D) ordinations were reliable.

Lack of any clear pattern between trail types and species was confirmed by PERMANOVA. No difference was found between *S. youngsoni* actual spool and control trails in composition (*Pseudo*-*F*_1,18_ = 0.09, *p* = 0.89) or size classes (*Pseudo*-*F*_1,19_ = 0.23, *p* = 0.81) of arthropod prey. Similarly, arthropod taxa and size classes did not vary between the actual and control tracks of lycosids (*Pseudo*-*F*_1,18_ = 0.46, *p* = 0.77 and *Pseudo*-*F*_1,18_ = 0.004, *p* = 0.91, respectively). Arthropod taxa were not dissimilar along the trails of *S. youngsoni* and lycosids (*Pseudo*-*F*_1,19_ = 1.50, *p* = 0.22), and no difference was found along trails of the two species-groups for arthropod size classes (*Pseudo*-*F*_1,19_ = 1.36, *p* = 0.28).

### 3.2. Temporal activity and overlap

**Figure 5.**
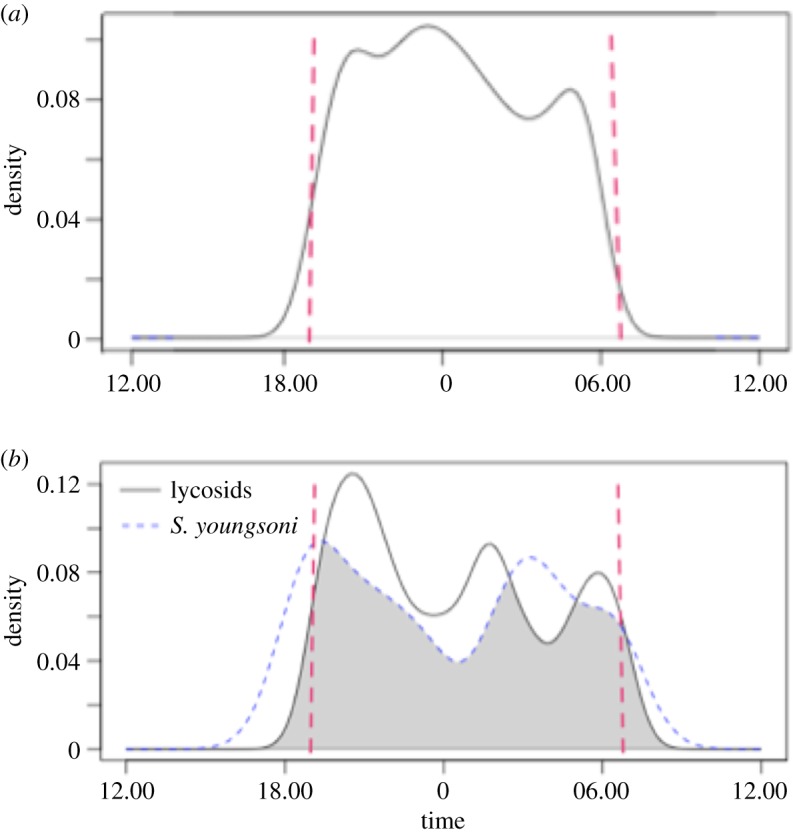
Fitted kernel density curves for (*a*) lycosid activity revealed through nightly spotlighting surveys, and (*b*) times of observation for lycosids (black solid line) and *Sminthopsis youngsoni* (blue dashed line) obtained from camera traps deployed at Main Camp, Simpson Desert, southwestern Queensland, between July and October 2016. Red dashed vertical lines represent the average times of dawn and dusk during spotlighting surveys and the camera deployment period. The coefficient of overlapping is the area under the lower of the two curves (grey shaded area) in (*b*).

Spotlighting surveys revealed that lycosids were active throughout the night, with a mean activity time of 00.19 h (95% CI 23.58–00.40 h). Activity peaked between 23.00 and 23.30 h and was least around 03.00–03.30 h (figure 5*a*).

Overall, 479 210 images were obtained from camera traps, taking over 70 h to process with approximately 1.6% of images containing fauna. A range of vertebrate and invertebrate species was detected, including 372 images (all confidence ratings included) of lycosids (electronic supplementary material, figure A1). These lycosids were captured solely from time-lapse images; i.e. lycosids did not trigger motion-capture. Both study species were completely nocturnal, with mean (±95% CI) nightly activity times of 00.11 (23.46–00.35) and 00.24 (22.02–02.46) for lycosids and *S. youngsoni,* respectively (electronic supplementary material, figure A2). Coefficient of overlapping in activity patterns of *S. youngsoni* and lycosids was 0.79 (figure 5*b*). This value was similar to an estimated mean of 0.76, and fell within the 95% confidence interval limits (0.61–0.87) calculated via bootstrapping.

### 3.3. Microhabitat use and overlap

In total, 26 *S. youngsoni* were spooled in 2016 (15 in July and 11 in October 2016) with an average spool length of 16.4 m (s.e. ± 0.97 m). The longest distance travelled was 30.7 m by a male (9.5 g) in July. Microhabitat use was determined for 20 lycosids during 2016 (12 in July and 8 in October), with an average trail length of 25.1 m (s.e. ± 3.04 m). The furthest distance travelled by an individual spider was 34.6 m.

**Figure 6.**
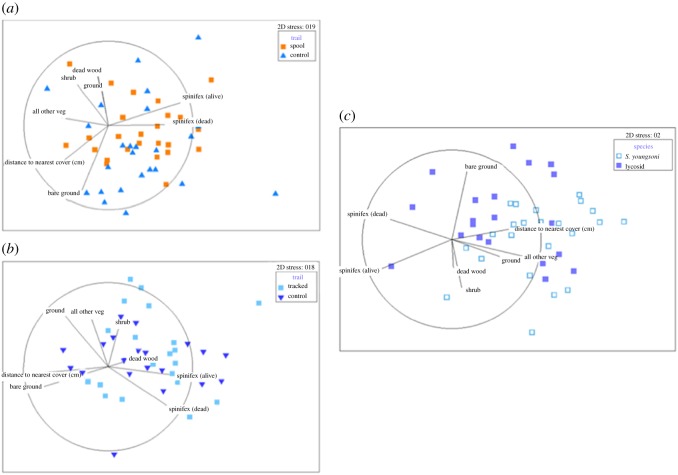
Non-metric multidimensional scaling ordination plots for habitat attributes along spool and control lines for (*a*) *Sminthopsis youngsoni*, (*b*) lycosids, and (*c*) microhabitat use between both species-groups from Main Camp, Simpson Desert, southwestern Queensland, in July and October 2016. Data were normalized and fourth-root transformed before ordination was performed using Euclidean distances. Stress values are less than 0.2, indicating that similarity between data points in 2D is reliable. Vectors for each habitat attribute were fitted to the ordination, with vector length proportional to the Spearman's correlation between the variable and the ordination, and direction indicating the gradient of each variable.

Non-metric MDS ordination found no clear separation between actual and control trails of either species (figure 6). Control trails for *S. youngsoni* appeared to cluster toward bare ground, while spool trails were more correlated with live and dead spinifex (figure 6*a*). Lycosid paths seemed to be associated with shrub and dead spinifex cover (figure 6*b*). A small degree of separation was apparent between *S. youngsoni* and lycosids, with the former associated with distance to nearest cover and the latter more strongly with bare ground and dead spinifex cover (figure 6*c*). Stress values were less than 0.2 for trail type for both species, signifying that 2D representation of datapoints was reliable. However, ordination between the two species revealed a Kruskal stress value of 0.2, which provides more marginal representation of the data.

PERMANOVA revealed that control trails were not dissimilar to actual spool trails within seasons for *S. youngsoni* (*Pseudo*-*F*_2,46_ = 1.24, *p* = 0.24). However, season did differ (*Pseudo*-*F*_1,46_ = 4.29, *p* < 0.001), with this component accounting for 99.6% of variation between groups. In comparison, PERMANOVA for lycosids revealed significant dissimilarity between both trail type nested within season (*Pseudo*-*F*_2,36_ = 2.08, *p* = 0.016) and season (*Pseudo*-*F*_1,36_ = 6.53, *p* < 0.001). PERMANOVA comparing microhabitat use between lycosids and dunnarts (control trails excluded) revealed dissimilarity between species nested within season (*Pseudo*-*F*_2,41_ = 3.31, *p* = 0.001), but showed season to be not significant (*Pseudo*-*F*_1,41_ = 1.20, *p* = 0.30). SIMPER revealed an average distance of 16.89 between these two species (the smaller the distance, the more similar the two species). Distance to nearest cover contributed the most to this difference (14.9%), followed by ground cover (13%). However, percentage contributions for all habitat variables were very similar (table 2).

**Table 2.** Similarity percentage (SIMPER) results for habitat variables measured along actual trails of *Sminthopsis youngsoni* and lycosids (*Lycosa* spp.). SIMPER was based on Euclidean distances, where greater distances indicate greater difference between the two species. The average squared distance was 16.89.

habitat variable	average value *S. youngsoni*	average value lycosids	average squared distance	squared distance/s.d.	% contribution	% cumulative contribution
distance to nearest cover (cm)	0.438	−0.547	2.51	0.80	14.89	14.89
ground^a^	0.265	−0.331	2.21	0.66	13.06	27.95
spinifex (dead)^a^	−0.280	0.350	2.18	1.01	12.90	40.85
all other vegetation^a^	0.236	−0.295	2.07	0.88	12.26	53.11
shrub^a^	−0.125	0.156	2.04	0.56	12.08	65.20
bare ground^a^	−0.140	0.175	1.97	0.65	11.65	76.84
spinifex (alive)^a^	−0.005	0.119	1.97	0.73	11.63	88.48
dead wood^a^	0.094	0.117	1.95	0.89	11.52	100.00

^a^Measured as % cover (table 1).

## 4. Discussion

In line with our initial prediction, *S. youngsoni* exhibited greater overlap in diet with lycosids than with miturgids. Additionally, a high degree of overlap was revealed in the timing of nightly activities of *S. youngsoni* and lycosids, as well as moderate overlap in their use of available microhabitats. Below, we examine in turn each aspect of this association.

### 4.1. Overlap in diet

There was more overlap in diet between *S. youngsoni* and lycosids, while weaker overlap in diet was observed between *S. youngsoni* and miturgids, thus supporting our first hypothesis that *S. youngsoni* would exhibit greater overlap in diet with lycosids than with other common invertebrate predators. Overlap values greater than 0.75 (i.e. 75% similarity) imply that two predators are consuming effectively the same prey taxa in similar proportions [26]. Hence, 83% similarity between lycosids and *S. youngsoni* signifies major dietary overlap and a strong case for designating these two taxa as members of the same dietary guild. Further support for this guild assignment derives from the overlap values obtained between *S. youngsoni* and miturgids (0.39), as well as the similarity between lycosids and miturgids. This latter result is particularly interesting as, although these spiders are more closely related and morphologically similar to each other than either is to *S. youngsoni*, their overlap in diet was only 68%. These observations reinforce ideas about guild definitions that group species on the basis of shared resources irrespective of taxonomic association [4,7,9].

The diversity of prey types consumed by all three predators was low, particularly for *S. youngsoni*. Limited information exists on the behaviour and ecology of spiders in arid Australia and, as such, these results provide valuable information on the diet of these arthropod predators. In comparison, knowledge of the diet of *Sminthopsis* spp. in arid environments is quite extensive [2,31,35,104]. However, our results contrast with the common view that *S. youngsoni* is a generalist insectivore that consumes a range of arthropods [31,35]. Instead, we show that *S. youngsoni* is more selective than previously thought in depredating lycosids, ants, and a small range of additional taxa. Indeed, the dietary diversity value obtained for *S. youngsoni* (0.18) is not dissimilar to that of several ‘facultative specialists’ (*sensu* [105]; see also [10,66,67]). Although our sample size was small, it seems likely that this species is a selective rather than a specialist forager.

Degree of trophic specialization, along with relative body size, is central in determining the direction and frequency of IGP [25]. For instance, IGP is more likely to arise in systems where distinctive size-structuring of populations occurs, with generalist predators larger than their subordinate prey [25,106]. Contrarily, however, competition and overlap are likely to be strongest between species that exhibit some degree of selection or specialization for a common prey type. In this study, there was more overlap in prey types consumed between the two predator-groups with the lowest dietary diversity, *S. youngsoni* and lycosids. These findings are consistent with previous research investigating dietary overlap and guild partitioning in desert skinks, where the greatest overlap of 93% was identified between two termite specialists *Ctenotus pantherinus* and *C. schomburgkii* [73].

Prey categories driving the strong overlap between lycosids and *S. youngsoni* were ants and spiders, including lycosids. This is particularly interesting as ants are generally not regarded as a substantial component in the diet of *S. youngsoni* [31,37]. A study on the diet of insectivorous marsupials in arid Australia found that although ants are commonly consumed by most species, they are avoided in relation to their availability [31]. As ants comprised over 90% of invertebrates captured along dunnart movement trails in our work, to consume ants in the same ratio as their local abundance, *S. youngsoni* would have to eat 90 times more ants than any other prey taxon. As this was not the case, the results suggest that dunnarts still forage selectively, but ants are nonetheless avoided.

Non-metric multidimensional scaling and PERMANOVA revealed no differences between trail types for both species-groups, implying that lycosids and dunnarts are not selecting paths with higher abundances of certain arthropod prey types or prey in particular size classes. This finding appears contrary to results from our direct observations that showed certain prey taxa to be consumed more often than others. This anomaly highlights potential biases associated with using invertebrate pitfall traps along foraging trails to assess prey availability. Firstly, no lycosids were caught in pitfall vials in this study. This may be attributable to the large eyes of these spiders. Lycosids have excellent night vision compared to other invertebrates, including miturgids that have only eight small eyes [34,36,90]. Hence, lycosids may be able to distinguish the depression or disturbance that pitfall traps create in the local environment and therefore avoid them (R. Raven 2017, personal communication). Additionally, the longer leg span of some individuals may preclude them from being caught in these traps. Lycosids have been captured using this method previously [28,32,37], but larger diameter pitfall traps nonetheless may be necessary in future studies examining availability of these spiders [107].

No significant difference was found between *S. youngsoni* and lycosids in terms of the arthropod taxa and prey size classes sampled along their actual foraging trails. Size is an important consideration when looking at prey overlap as, although species pairs may eat the same prey taxa, competition can be reduced if the two species target different-sized prey [43]. Overlap values for prey sizes derived from direct observations indicated that there was greater similarity in prey sizes consumed by *S. youngsoni* and lycosids (68%) than by *S. youngsoni* and miturgids (57%); however, 91% similarity was observed between the two spider families. Previous research has found a positive relationship between predator body size and the size of prey consumed [35,57], and thus these overlap values may reflect the closer similarity in body size between the two spider groups. Dunnarts will eat prey of almost any size up to their own body mass [31], but it was interesting to note also that lycosids can eat prey at least three times their size. For example, a 9 mm-long lycosid was observed preying on a mantid 30 mm long (T. Potter 2016, personal observation).

### 4.2. Overlap in temporal activity

Both lycosids and *S. youngsoni* were almost completely nocturnal, although *S. youngsoni* appeared to emerge earlier (approx. 15.00 h) and remain active until later in the morning (approx. 09.30 h) compared to lycosids (approx. 17.00 h–approx. 08.00 h). When examining lycosid activity from spotlighting surveys, a main peak was evident around midnight, followed by a decline in activity during the early hours of the morning and then a rise in activity again just before dawn. An analogous decline in activity was revealed from camera images. Another interesting comparison is that a second dip in activity was recorded earlier in the evening for both the spotlighting and camera activity curves. This dip occurred at 22.00 h and was only minor from direct observations, but camera traps revealed this decline to occur at 23.00 h, with a more pronounced trough apparent.

In comparison, activity of *S. youngsoni* was distinctly bimodal with an initial peak around dusk (18.30 h), a secondary peak in the early hours of the morning (approx. 04.00 h) and a marked trough just after midnight. This curve mirrors activity patterns previously described for this species, and directly observed during this study, where the dunnarts are more active during the warmer, earlier phase of the night and then enter torpor during the latter, cooler part of the night and early morning [53,108]. When assessing the activity of lycosids and *S. youngsoni*, some degree of temporal partitioning is apparent, with the peak in lycosid activity corresponding with when dunnarts are entering torpor (i.e. ∼midnight). This moderate temporal dissociation may reduce encounter rates and predation on lycosids [43,109], thereby enabling coexistence of these two taxa [110]. Although bootstrapping suggested temporal overlap between the two taxon-groups was no different from random, the calculated similarity in activity times, 76%, suggests that individuals of both groups would encounter each other frequently.

### 4.3. Overlap in microhabitat use

No difference was observed in the use of different microhabitats between actual spool and control trails for *S. youngsoni*; however, season did have an effect. This indicates that although the availability of particular microhabitats may have changed between seasons, *S. youngsoni* still selected microhabitats relative to their availability. In contrast, trail type differed with season for lycosids, suggesting that lycosids exhibited a preference for certain microhabitats irrespective of their relative abundance.

An ostensible difference was found in microhabitat use between *S. youngsoni* and lycosids, with *S. youngsoni* influenced by distance to cover and lycosids appearing to prefer areas with less bare ground and more spinifex cover. However, the contributions of distance to cover and amount of dead spinifex to this perceived difference were only marginal, i.e. approximately 15% and approximately 13%, respectively. This indicates that, although there is a disparity in habitat use between these two taxa, the difference is subtle. Lycosids may select areas with greater cover as refuges to minimize predation by the dominant *S. youngsoni.* Previous research has demonstrated that lycosids are able to detect vibratory, visual and chemotactile (i.e. faeces, silk) cues left by hetero- and conspecifics and assimilate these into decisions relating to predator avoidance [111,112]. Hence, lycosids may be able to detect when *S. youngsoni* is present and therefore avoid them. In circumstances where intense competition is likely, there is a strong selective advantage for species to exhibit divergence in their respective niches [110]. Species will typically segregate along axes of diet and habitat use before exhibiting temporal partitioning as a means to facilitate coexistence [110]. Consequently, small scale spatial avoidance of a larger predator by a subordinate species is often associated with IGP [25,109]. For example, foxes avoid sites with dingoes [19], wolves restrict the abundance and home ranges of coyotes [16,109,113], and Egyptian mongooses evade areas where densities of Spanish lynx are high [114].

### 4.4. Future directions

Due to the highly stochastic climatic conditions in central Australia, and the resultant fluctuations in food and microhabitat availability, the degree of overlap in diet and microhabitat use between *S. youngsoni* and lycosids is likely to change after increases in productivity from rainfall-initiated resource-pulses (‘booms’) compared to dry conditions (‘busts’). Consequently, the strength of competition and potential IGP between these species-groups may weaken or intensify under different circumstances [25]. For instance, when prey is scarce, antagonistic encounters may escalate as predators searching for similar prey are likely to forage in the same habitats, or dominant predators may alter their diet to include guild members [14,18,25]. As *S. youngsoni* was observed selectively consuming lycosids during a very wet and productive year when prey were likely to be plentiful, selective depredation of lycosids may intensify still further during drought periods. Conversely, as *S. youngsoni* can be classed as a ‘facultative specialist’, selective depredation of lycosids might weaken during drought conditions. The premise here is that predators can afford to specialize when food is abundant, but benefit more from being opportunistic and generalized in their diet when prey is scarce [105,115,116]. Intriguingly, Estrada [28] observed the selective targeting of lycosids under varied conditions, suggesting that IGP persists during both boom and bust periods.

Molecular techniques, including polymerase chain reaction-based gut content analysis, have been used previously to examine the diet of predators based on identification of DNA from arthropod prey in digested gut material (see review by [117]). However, these studies predominantly used laboratory-fed predators, with few studies applying this technique to field-based experiments [118,119]. The application of this technique could greatly enhance further explorations of the topic of IGP. Currently, the major drawback is the absence of a catalogue containing the DNA sequences of potential invertebrate prey with which to compare the sequences extracted from the gut of each predator. Overcoming this shortfall may occur in the future, but it would be quite labour-intensive, costly and challenging.

## 5. Conclusion

The hypothesis that *S. youngsoni* will exhibit greater overlap in diet with lycosids than with other common invertebrate predators was supported. Despite small sample sizes, similarity between these two species-groups was 83%, signifying major dietary overlap and a strong case for assignment to the same guild. Additionally, although there was some temporal partitioning, a high degree of overlap (79%) was identified in diel activities of lycosids and *S. youngsoni*. Results for microhabitat use were more ambiguous, with both species using the same microhabitats in the study area, but in different proportions. Lycosids exhibited a preference for greater spinifex cover and less bare ground, suggesting that spinifex may be used as a refuge to minimize predation by *S. youngsoni* and other threats. Taken together, these findings suggest there is reasonably strong potential for competition and IGP to occur between lycosids and *S. youngsoni*. If confirmed, this would represent the most taxonomically disparate example of IGP that has been reported.

## Supplementary Material

Appendix Tables and Figures

## Data accessibility

Supporting data have been published through AEKOS as:

Potter TI, Greenville AC, Dickman CR. 2017 Availability of invertebrate prey for micro-carnivores, Version 1. doi:10.4227/05/5a17a9ab8652b. ÆKOS Data Portal, rights owned by University of Sydney.

Potter TI, Greenville AC, Dickman CR. 2017 Temporal activity of wolf spiders and dunnarts in the Simpson Desert, Version 1. doi:10.4227/05/5a167887d329a. ÆKOS Data Portal, rights owned by University of Sydney.

Potter TI, Greenville AC, Dickman CR. 2017 Direct observations of foraging wolf spiders and dunnarts, Version 1. doi:10.4227/05/5a151e727fb2f. ÆKOS Data Portal, rights owned by University of Sydney.

Potter TI, Greenville AC, Dickman CR. 2017 Microhabitat selection by wolf spiders and dunnarts, Version 1. doi:10.4227/05/5a1f43d3542e5. ÆKOS Data Portal, rights owned by University of Sydney.
