# Supplementary material for: Assessing the potential for intraguild predation among taxonomically disparate micro-carnivores: marsupials and arthropods
Source: R Soc Open Sci. 2018 May 2;5(5):171872. doi: 10.1098/rsos.171872 (PMC5990775; doi:10.1098/rsos.171872)
Supplement: Appendix Tables and Figures [file rsos171872supp1.docx]

Appendix

**Table A1.** Results for PERMDISP testing homogeneity of multivariate dispersions between prey taxa and size classes of arthropods captured in invertebrate pitfall traps. These traps were deployed along actual and control trails of *Sminthopsis youngsoni* and lycosids (*Lycosa* spp.) at Main Camp, Simpson Desert, south-west Queensland during October 2016.

| **Species** | **Arthropod Data** | **d.f.** | ***F* statistic** | ***P*** |
| --- | --- | --- | --- | --- |
| *S. youngsoni* | Prey Taxa | 1, 19 | 2.53 | 0.14 |
|  | Size Class | 1,19 | 9.20 | **0.005** |
| Lycosids | Prey Taxa | 1,18 | 0.02 | 0.90 |
|  | Size Class | 1,18 | 5.87 | **0.042** |

*Bold *P* values indicate significance at α <0.05

**Table A2.** Results from PERMDISP testing homogeneity of multivariate dispersions for microhabitat data between trail type (actual or control) and season (winter or spring) for *Sminthopsis youngsoni* and lycosids (*Lycosa* spp.) tracked at Main Camp, Simpson Desert, south-west Queensland, in 2016.

| **Species** | **Data** | **d.f.** | ***F* statistic** | ***P*** |
| --- | --- | --- | --- | --- |
| *S. youngsoni* | Trail type | 1, 48 | 2.32 | 0.17 |
|  | Season | 1,48 | 0.82 | 0.41 |
| Lycosids | Trail type | 1,38 | 0.20 | 0.66 |
|  | Season | 1,38 | 7.19 | **0.016** |

*Bold *P* value indicates significance at α <0.05


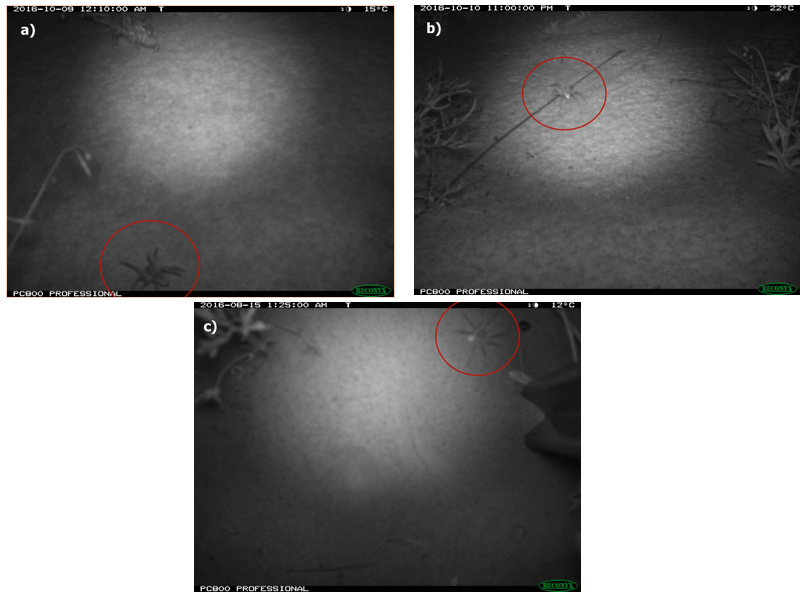


**Figure A1.** Photographs of wolf spiders (Lycosidae) taken using remote-sensing cameras deployed at Main Camp, Simpson Desert, south-western Queensland, between July and October 2016. Two camera positions were employed to maximise capture success of the study species, i.e. 45° angle (a & b) or vertical (c). Lycosids were distinguished from prowling spiders (Miturgidae) by the raised carapace (evident in image a) and shape of the prosoma, which is more rounded compared to miturgids.


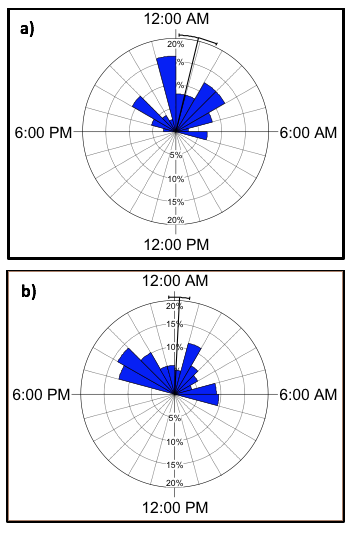


**Figure A2.** Diel activity patterns of a) *Sminthopsis youngsoni* and b) lycosids (*Lycosa* spp.) extracted from camera images deployed at Main Camp, Simpson Desert for 98 days (2352 h in the field) between July and October 2016. Vectors (black line) represent mean activity times with 95% confidence intervals, while the radius of each wedge is equal to the frequency of observations (as a %) within that time period.
